# The EMSY threonine 207 phospho-site is required for EMSY-driven suppression of DNA damage repair

**DOI:** 10.18632/oncotarget.14637

**Published:** 2017-01-13

**Authors:** Petar Jelinic, Laura A. Eccles, Jill Tseng, Paulina Cybulska, Monicka Wielgos, Simon N. Powell, Douglas A. Levine

**Affiliations:** ^1^ Laura and Isaac Perlmutter Cancer Center, Division of Gynecologic Oncology, Department of OB/GYN, NYU Langone Medical Center, New York, USA; ^2^ Departments of Radiation Oncology and Molecular Biology, Memorial Sloan Kettering Cancer Center, New York, USA; ^3^ Department of Surgery, Memorial Sloan Kettering Cancer Center, New York, USA

**Keywords:** EMSY, BRCAness, PKA, BRCA2, DNA repair

## Abstract

*BRCA1* and *BRCA2* are essential for the repair of double-strand DNA breaks, and alterations in these genes are a hallmark of breast and ovarian carcinomas. Other functionally related genes may also play important roles in carcinogenesis. Amplification of *EMSY*, a putative BRCAness gene, has been suggested to impair DNA damage repair by suppressing BRCA2 function. We employed direct repeat GFP (DR-GFP) and RAD51 foci formation assays to show that EMSY overexpression impairs the repair of damaged DNA, suggesting that EMSY belongs to the family of BRCAness proteins. We also identified a novel phospho-site at threonine 207 (T207) and demonstrated its role in EMSY-driven suppression of DNA damage repair. *In vitro* kinase assays established that protein kinase A (PKA) directly phosphorylates the T207 phospho-site. Immunoprecipitation experiments suggest that EMSY-driven suppression of DNA damage repair is a BRCA2-independent process. The data also suggest that EMSY amplification is a BRCAness feature, and may help to expand the population of patients who could benefit from targeted therapies that are also effective in *BRCA1/2*-mutant cancers.

## INTRODUCTION

Impairment of homology-directed repair (HDR) of double-strand breaks is a major alteration in many cancers [1]. Extensive research throughout the last few decades has revealed that mutations in *BRCA1* and *BRCA2*, encoding essential proteins in the HDR pathway, are a critical component of HDR impairment in hereditary and sporadic breast and ovarian carcinomas [2]. Emerging evidence suggests that the HDR pathway is altered in additional sporadic cancers with intact BRCA genes [3, 4]. These alterations, referred to as BRCAness features, are thought to reflect the properties of BRCA-mutant cancers, including improved treatment response to certain types of therapies. Thus, patients with BRCAness features may benefit from therapies known to be effective in patients bearing cancers with *BRCA1* and *BRCA2* mutations.

**Figure 1:**
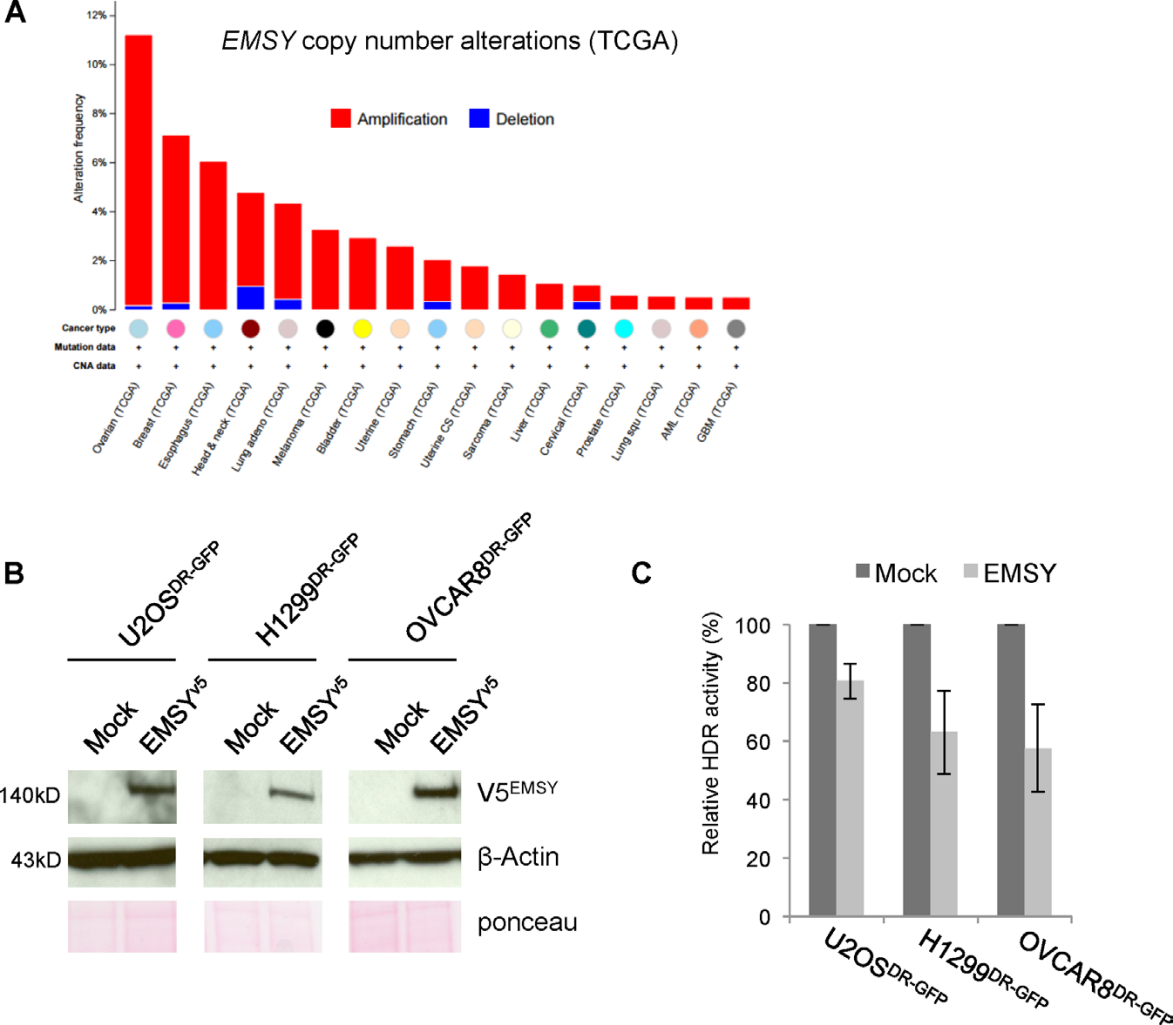
Full-length EMSY overexpression suppresses HDR activity (**A**) Pan-cancer copy number alterations in *EMSY*. Percentages are from TCGA studies using data at cbioportal.mskcc.org (downloaded on March 7, 2016) [39, 40]. (**B**) Full-length V5-tagged EMSY overexpression in U2OS^DR-GFP^, H1299^DR-GFP^, and OVCAR8^DR-GFP^ cells assessed by western blot. Overexpressed EMSY is detected with the V5 antibody (V5^EMSY^). Anti-β-Actin and ponceau are loading controls. (**C**) DR-GFP assay measuring HDR activity in EMSY-overexpressing cells. Percentages of GFP-positive EMSY-overexpressing cells were normalized to mock controls (empty vector transfected) to obtain relative HDR activity.

Amplification of *EMSY*, a putative BRCAness feature, has been proposed to mimic the BRCA2-mutant phenotype [5]. *EMSY* maps to chromosome 11q13.5, hence its official name c11orf30 (chromosome 11 open reading frame 30). This locus is frequently amplified in ovarian and breast cancers [6, 7]. *EMSY* is reported to be amplified in 8–14% of breast cancers [5, 8, 9] and up to 18% of ovarian cancers [5, 10, 11]. A recent comprehensive genomic survey of multiple cancer types by The Cancer Genome Atlas (TCGA) revealed that *EMSY* is predominantly amplified in high-grade ovarian cancer [12] and invasive breast carcinoma [13], ~11% and ~7%, respectively (Figure 1A).

EMSY was initially described as a BRCA2-interacting protein. It has been hypothesized that *EMSY* amplification mimics the BRCA2-mutated state through direct interaction with BRCA2, resulting in suppression of protein function [5]. It has an evolutionarily conserved EMSY N-terminal (ENT) domain that is thought to bind directly to the N-terminal region of BRCA2. Several groups have proposed EMSY's role in maintenance of chromosomal stability and HDR [5, 14, 15]; however, a major limitation of these prior studies has been the assessment of cells with overexpression of only the truncated form of EMSY. It has been difficult to overexpress the full-length EMSY, and to date, the impact of full-length EMSY on HDR has not been addressed. The direct interaction between EMSY and BRCA2 leading to impaired HDR has not been demonstrated.

EMSY has also been shown to have other roles unrelated to HDR mechanisms. Early studies described EMSY acting as a transcription factor *via* its interaction with chromatin-associated proteins BS69 and HP1-β, proposing a role for EMSY in the suppression of target genes [5, 16, 17]. Phosphorylation by AKT1, a protein kinase activated by phosphoinositide-3-kinase (PI3K), has recently been suggested to be important in EMSY's role as a transcription factor [18]. However, the relevance of EMSY phosphorylation in the context of HDR suppression has not been studied.

Here, we assess the impact of full-length EMSY overexpression on HDR activity in multiple cell lines. We also describe a novel phospho-site at threonine 207 (T207) that is required for the EMSY overexpression-driven suppression of HDR. Finally, we identify a kinase that targets this phospho-site and discuss the effects of EMSY phosphorylation on the HDR pathway in a BRCA2-independent manner.

## RESULTS

### Full-length EMSY overexpression impairs HDR activity

We successfully overexpressed full-length EMSY in our model cell lines (Figure 1B), thus allowing us to test whether full-length protein overexpression affects HDR. To address this, we utilized a well-established direct repeat GFP (DR-GFP) reporter assay [19]. This assay takes advantage of a stably integrated GFP cassette that expresses GFP only upon repair of I-SceI endonuclease-induced DNA damage. GFP expression is used as a read-out for the presence of a functional HDR pathway. We assessed HDR activity in three cell lines with stably integrated DR-GFP reporter: U2OS^DR-GFP^, H1299^DR-GFP^, and OVCAR8^DR-GFP^. The cells were co-electroporated with I-SceI endonuclease-expressing vector and either empty vector (mock) or EMSY-expressing plasmid. The transfected cells were incubated for 48 h to allow repair of I-SceI-induced DNA damage. GFP-positive cells were then quantified with flow cytometry. All three cell lines showed a decrease in HDR activity upon EMSY overexpression (Figure 1C). The EMSY-overexpressing U2OS^DR-GFP^ cell line showed an approximate 20% decrease in HDR activity. In H1299^DR-GFP^ and OVCAR8^DR-GFP^ cell lines, HDR activity decreased more prominently upon EMSY overexpression (~37% and ~43%, respectively). We also tested if depletion of EMSY in *EMSY*-amplified cells would affect HDR activity. We knocked down EMSY in OVCAR3, an ovarian cancer cell line with *EMSY* amplification, and measured RAD51 foci formation upon treatment with camptothecin (CPT), an inhibitor that targets DNA topoisomerase I resulting in collapse of the replication fork and DNA double-strand breaks (Supplementary Figure 1). The formation of RAD51 foci after DNA damage reflects the assembly of protein complexes necessary for DNA damage repair [20]. We did not observe any significant changes in HDR activity. Altogether, these findings suggest that EMSY overexpression impairs HDR activity.

### Threonine 207 is required for EMSY overexpression-driven HDR suppression

**Figure 2:**
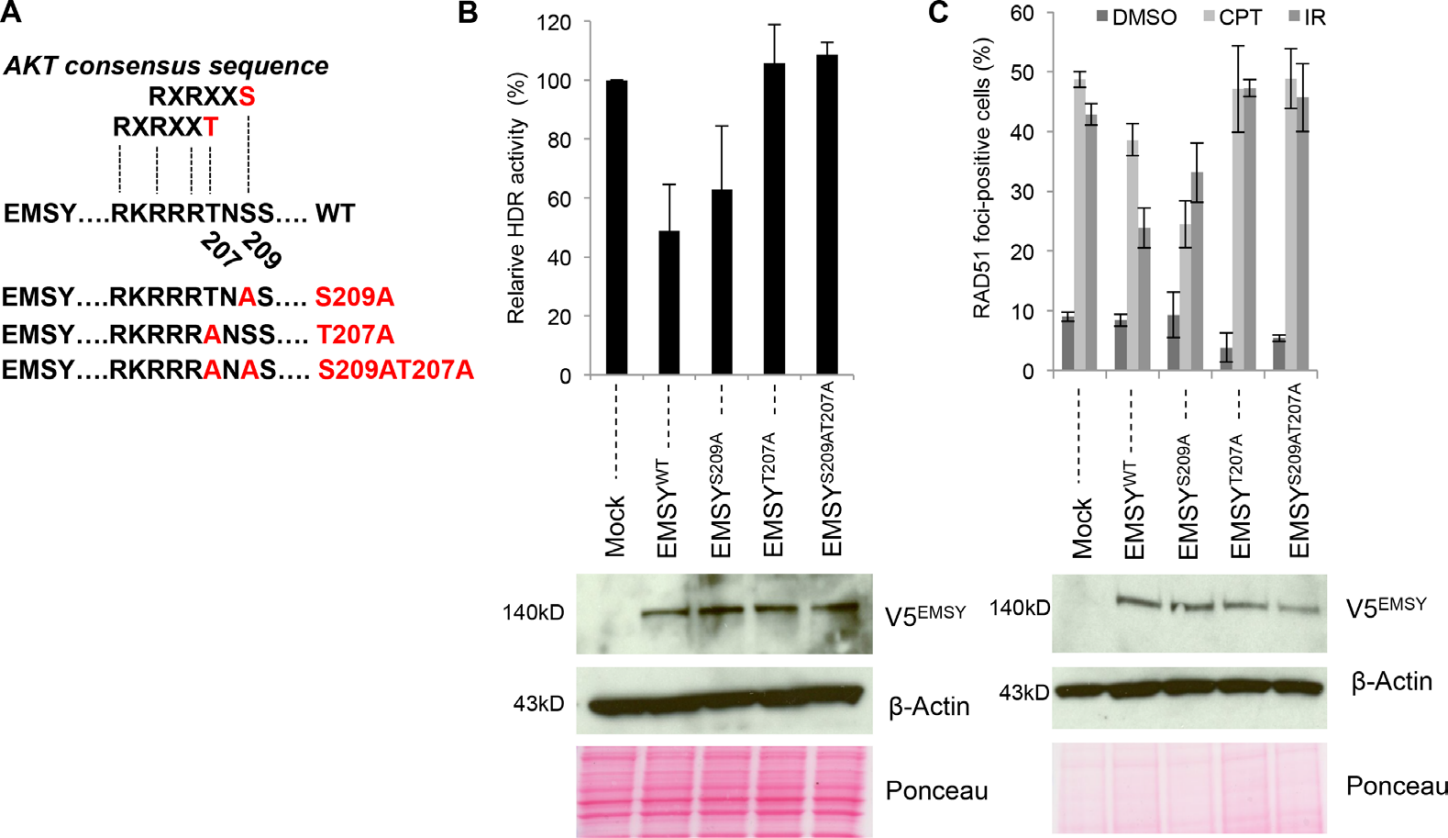
The EMSY T207 phospho-site is required for the EMSY-driven suppression of HDR activity (**A**) Schematic presentation of the AKT consensus sequence and EMSY mutants. (**B**) DR-GFP assay measuring HDR activity in OVCAR8^DR-GFP^ overexpressing wild type and mutant EMSY. Percentages of GFP-positive EMSY-overexpressing cells were normalized to mock controls (empty vector transfected) to obtain relative HDR activity. Panels below in (B) and (**C**) are western blots showing overexpression of wild type and mutant EMSY. Overexpressed EMSY is detected with the V5 antibody (V5^EMSY^). Anti-β-Actin and ponceau are loading controls. (C) RAD51 foci formation assay in OVCAR8 cells overexpressing wild type and mutant EMSY. To induce DNA damage, cells were treated with either 4 μM camptothecin (CPT) or irradiation (10 Gy). DMSO-treated cells were used as a control for detecting the baseline RAD51 foci formation. Cells with five or more RAD51 foci were counted as positive.

It is known that EMSY is phosphorylated at serine 209 (S209) by the serine/threonine protein kinase AKT1 [18]. The importance of the S209 phospho-site for EMSY's role as a transcription factor has been proposed; however, the importance of AKT1-driven EMSY phosphorylation in the context of HDR has not been addressed. Several studies have suggested a direct relationship between AKT1-targeted phosphorylation and HDR [21, 22], and we tested whether the S209 phospho-site is necessary for EMSY overexpression-driven HDR impairment. We constructed an EMSY S209A mutant and employed the DR-GFP assay in OVCAR8^DR-GFP^ cells. Cells overexpressing EMSY-S209A mutant and those overexpressing wild type EMSY had similar decreases in HDR activity (Figure 2B), suggesting that the S209 phospho-site is not necessary for EMSY overexpression-driven HDR impairment.

Closer examination of EMSY's protein sequence identified another potential AKT-targeting phospho-site at threonine 207 (T207). Aside from S209, T207 is the only other amino acid that falls within the AKT-phospho-consensus sequence, RXRXX(T/S). To further explore this putative phospho-site, we created additional EMSY mutants (Figure 2A and Supplementary Figure 2). First, we tested whether the T207 putative phospho-site was necessary for EMSY-driven HDR impairment. We again employed the DR-GFP assay in OVCAR8^DR-GFP^ cells (Figure 2B). Wild type and mutant full-length EMSYs were co-electroporated with I-SceI endonuclease, and after 48 hours, GFP expression was measured. Compared to the wild type, cells overexpressing EMSY-S209A mutant showed minimal difference in HDR impairment (~52% versus ~37%, respectively) when normalized to the control cells (vector-only-transfected). However, overexpression of either mutant T207A or double-mutant T207AS209A EMSY did not result in reduced HDR impairment, and HDR activity was comparable to the control cells (~106% and ~108%, respectively). This suggests that EMSY's threonine 207 site is necessary for the EMSY overexpression-driven HDR impairment.

To confirm these findings, we tested HDR activity in EMSY wild type and mutant-overexpressing OVCAR8 cells using a RAD51 foci formation assay (Figure 2C and Supplementary Figure 3). To induce DNA damage, OVCAR8 cells overexpressing wild type or mutant EMSY were either irradiated (10Gy) or treated with 4 μM CPT. CPT-treated mock cells (vector-only-transfected) had ~49% RAD51-foci-positive cells. Upon EMSY wild type overexpression, the percentage of RAD51-foci-positive cells was ~39%, demonstrating an approximate 20% decrease in HDR activity. Surprisingly, CPT-treated S209A-overexpressing cells had even fewer RAD51-foci-positive cells (~25%), exhibiting nearly a 50% decrease in HDR activity compared with the control. In contrast, the percentages of RAD51-foci-positivity in CPT-treated T207A- and T207AS209A-overexpressing cells were comparable with the control, ~47% and ~49% versus ~49%, respectively, confirming the dependence on the T207 phospho-site observed by the DR-GFP assay.

Irradiated control cells were ~43% RAD51-foci-positive. EMSY wild type overexpressing cells showed a decrease to ~24% in RAD51-foci-positivity, indicating an approximate 44% decrease in HDR activity compared with the control. Overexpression of S209A resulted in ~33% RAD51-foci-positive cells upon irradiation, reflecting an approximate 23% decrease in HDR activity. T207A- and T207AS209A-overexpressing cells had slightly higher percentages of RAD51-foci formation (~47% and ~45%, respectively) compared with control cells, suggesting no HDR activity impairment. Taken together, these data support the conclusion that the putative T207 phospho-site is required for EMSY overexpression-driven suppression of HDR activity.

### EMSY T207 phospho-site is targeted by protein kinase A (PKA)

**Figure 3:**
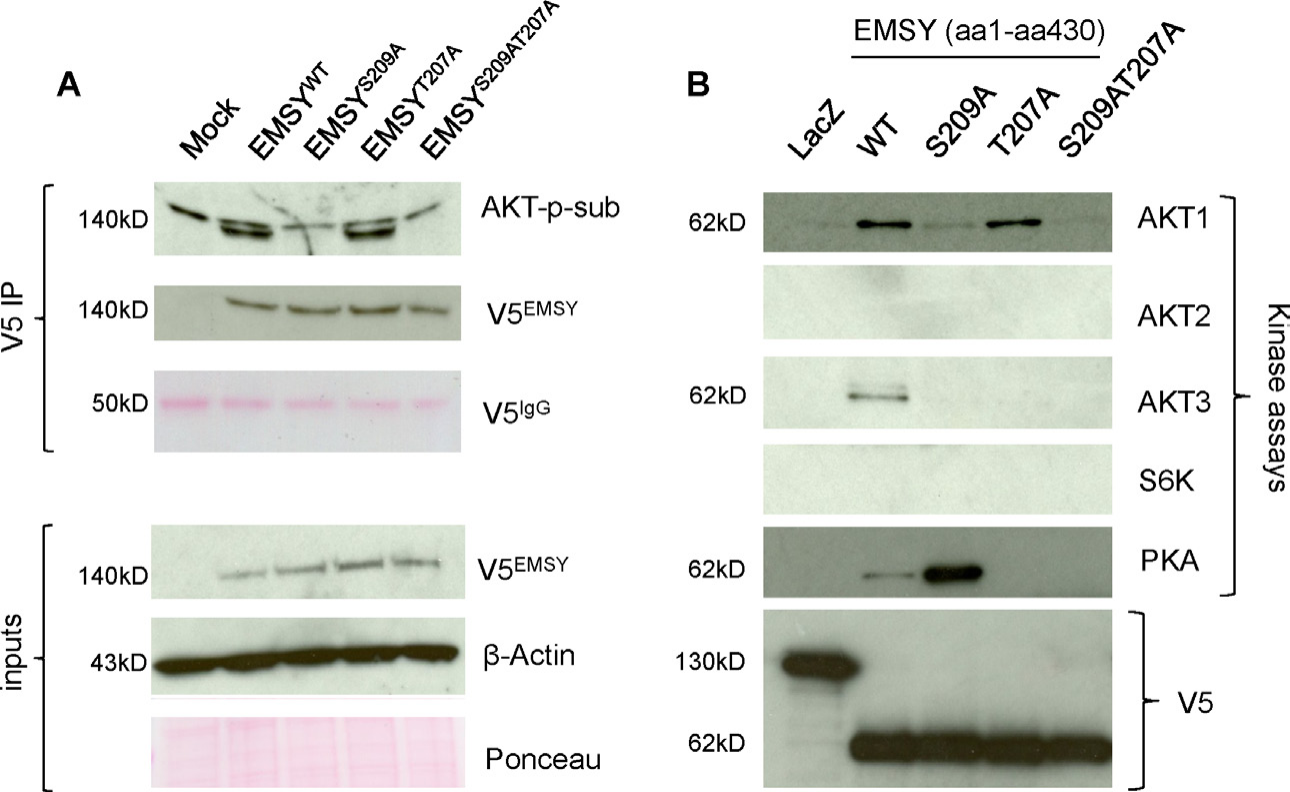
The EMSY T207 phospho-site is phosphorylated by PKA (**A**) EMSY-S209, but not EMSY-T207 is a target of AKT phosphorylation. OVCAR8 EMSY-overexpressing cell lysates were immunoprecipitated with V5 antibody and blotted with AKT-phosphosubstrate (AKT-p-sub) to detect AKT-targeted phosphorylation (upper bands are non-specific). The three upper and lower panels represent V5 immunoprecipitation (V5 IP) and starting material (inputs), respectively. Overexpressed EMSY, wild type and mutants, are detected with the V5 antibody (V5^EMSY^). The IgG heavy chain (V5^IgG^) ponceau staining served as a V5 immunoprecipitation loading control. Anti-β-Actin and ponceau are loading controls for input materials. (**B**) Western blot showing *in vitro* kinase assays using truncated V5-tagged N-terminal EMSY (aa1-aa430) expressed in *E. Coli*. EMSY wild type and mutants were reacted with the kinases shown. The AKT1, AKT2, AKT3, and S6K kinase reactions were probed with the AKT-phosphosubstrate antibody. The PKA kinase reaction was probed with the PKA-phosphosubstrate antibody. LacZ-expressing vector was used as a negative control. Equal amounts of LacZ and EMSY wild type and mutants are detected with the V5 antibody.

After confirming the importance of the T207 site in EMSY-driven HDR impairment, we tested T207 to see if it is a genuine phospho-site and if AKT1 is the targeting kinase. We transfected OVCAR8 cells with wild type and mutant full-length EMSY as previously described [18]. Following a 24-h incubation period, the cell lysates were immunoprecipitated with V5 antibody. The immunoprecipitates were probed with AKT-phosphosubstrate antibody that preferentially recognizes phospho-Ser/Thr preceded by lysine/arginine at positions -5 and -3. This antibody is not specific for phosphorylated EMSY and may show some cross-reactivity with other peptides containing Ser/Thr sites; however, V5-immunoprecipitation ensures the EMSY-phosphorylation-specific detection and minimizes a possibility of non-specificity. Figure 3A shows that EMSY is phosphorylated by AKT specifically at S209, confirming published data [18]. However, these data also suggest that the T207 putative phospho-site is not targeted by AKT.

To seek other potential kinases that specifically target the T207 putative phospho-site, we utilized a bioinformatic group-based prediction system (http://gps.biocuckoo.org). As expected, among top candidates were the AKT kinases: AKT1, AKT2, and AKT3. Two additional candidates were c-AMP-dependent PKA and ribosomal S6 kinase (S6K) (for the consensus sequences see Supplementary Figure 4).

To validate these predictions, we performed *in vitro* kinase assays (Figure 3B). Using Invitrogen's Champion pET Directional TOPO expression kit, we sub-cloned wild type and mutant N-terminal EMSYs (aa1-aa430) into pET101 inducible vector for expression in *E. coli*. As a negative control, we used LacZ-expressing pET101 plasmid. After induction with 1mM IPTG, the proteins were purified using nickel beads and eluted in a buffer containing 250 mM imidazole. The eluted proteins were then used for the kinase assays. After 30 min of incubations with corresponding kinases, EMSY proteins were resolved by SDS-PAGE and blotted with specific antibodies. The above-mentioned AKT-phosphosubstrate antibody was used in AKT1, AKT2, and AKT3 kinase assays. Since there was no commercially available S6K-phopsohsubstrate antibody and the S6K consensus motif for substrate R/KXXRXXS/T is recognized by the AKT-phosphosubstrate antibody, we used this antibody for the S6K kinase assay. The PKA-phosphosubstrate antibody that preferentially recognizes the RRXS/T motif was used for the PKA kinase assay. Although, these antibodies are not specific for phospho-EMSY detection, the purified EMSY is the only targeting protein in kinase assays excluding a possibility of false detection. The *in vitro* kinase experiments confirmed that AKT1 phosphorylates S209 but not T207. Furthermore, our results suggest that neither AKT2 nor S6K phosphorylates EMSY. AKT3, however, appears to phosphorylate EMSY at both the S209 and S207 phospho-sites. Finally, among those tested, PKA was the only kinase that specifically targeted the T207 phospho-site. Phosphorylation at the 207 phospho-site was more prominent in the S209A mutant compared with the wild type EMSY. Taken together, we demonstrate that T207 is a genuine phospho-site targeted by at least two kinases - AKT3 and PKA.

### PKA expression levels in cells affect EMSY phosphorylation

**Figure 4:**
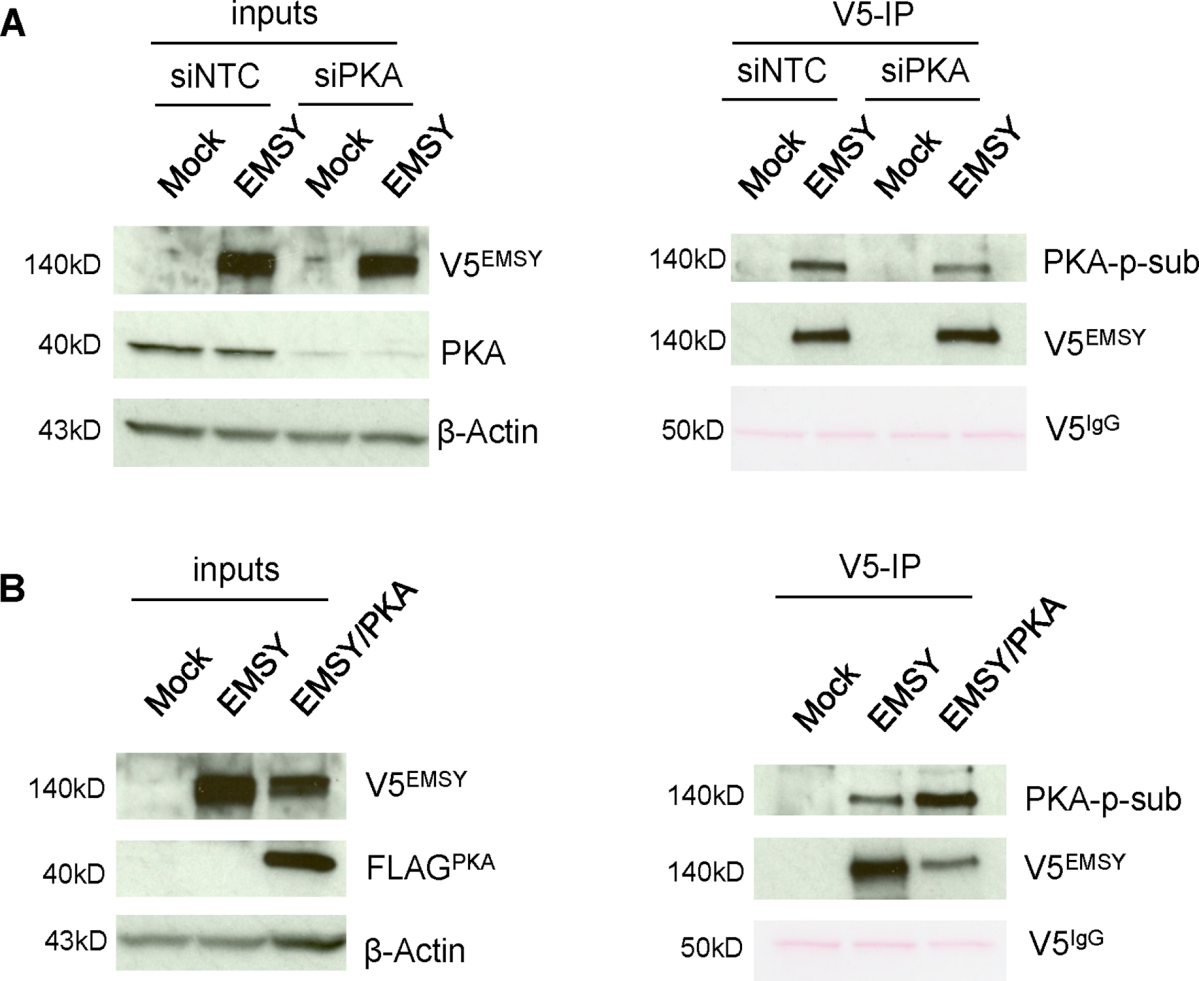
PKA expression levels affect EMSY phosphorylation in 293T cells (**A**) EMSY-overexpressed and/or PKA-depleted 293T cell lysates were immunoprecipitated with V5 antibody and blotted with PKA-phosphosubstrate (PKA-p-sub) antibody. The left side panels demonstrate EMSY overexpression and PKA knockdown by siRNA. The right panels show V5-immunoprecipitation (V5-IP) and detection of PKA-targeted EMSY phosphorylation using the PKA-p-sub antibody. Overexpressed EMSY is detected with the V5 antibody (V5^EMSY^). (**B**) EMSY and PKA co-expressed 293T cell lysates were immunoprecipitated with V5 antibody and blotted with PKA-p-sub. The left panels show EMSY and FLAG-tagged PKA overexpression. The right panels show V5-immunoprecipitation (V5-IP) and detection of PKA-targeted EMSY phosphorylation using the PKA-p-sub antibody. Overexpressed PKA is detected with FLAG (FLAG^PKA^) antibody. For (A) and (B) IgG heavy chain (V5^IgG^) ponceau staining and anti-β-Actin served as loading controls.

Our data suggest that the EMSY T207 phospho-site is required for EMSY overexpression-driven HDR impairment. We thus decided to focus on PKA, the only kinase tested that specifically targets EMSY's T207 phospho-site. To further confirm PKA-targeted EMSY phosphorylation, we manipulated 293T cells for depletion or overexpression of PKA (Figure 4). For knockdown experiments, 293T cells were electroporated with either non-targeting control siRNA (siNTC) or siPKA and seeded overnight. After 24 h, the cells were transfected with either empty vector (mock) or full-length EMSY expressing plasmid using FuGene reagent. The next day, cell lysates were prepared and proteins were immunoblotted. Upon PKA knockdown, EMSY phosphorylation was decreased (Figure 4A). However, EMSY phosphorylation was significantly increased upon EMSY and PKA co-expression (Figure 4B). We also noted that when EMSY was co-expressed with PKA, EMSY levels were decreased. Taken together, we confirmed our *in vitro* kinase assay data and showed that EMSY is phosphorylated by PKA. Further validation by PKA depletion or overexpression in EMSY-overexpressing cells showed that PKA-targeted EMSY phosphorylation decreased or increased in an inversely proportional manner.

### EMSY phosphorylation increases in cells treated with forskolin (PKA activator) and decreases in cells treated with H-89 (PKA inhibitor)

**Figure 5:**
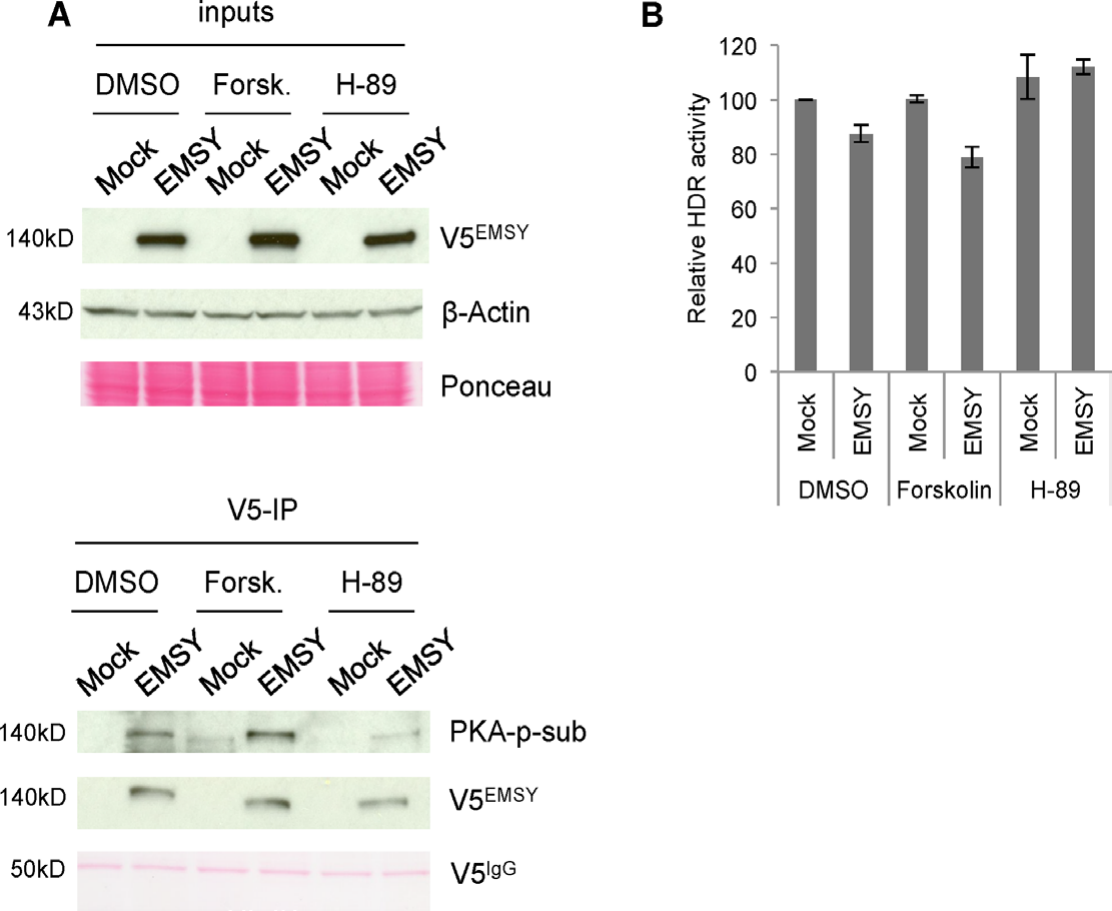
PKA activity is proportional to phosphorylation of EMSY and HDR activity in EMSY-overexpressing cells (**A**) EMSY-overexpressed 293T cells were treated with DMSO, the PKA activator forskolin, or the PKA inhibitor H-89. The left panels show the input lysates. The right panels show V5-immunoprecipitaions (V5-IP) and detection of EMSY phosphorylation with the PKA-phosphosubstrate (PKA-p-sub) antibody. Overexpressed EMSY is detected with the V5 (V5^EMSY^) antibody. Ponceau staining and anti-β-Actin served as loading controls. (**B**) DR-GFP assay measuring HDR activity in OVCAR8^DR-GFP^ overexpressing wild type EMSY. The cells are treated with DMSO, forskolin, or H-89. HDR activity was measured 24 h post-transfection and post-treatment. All data are normalized to DMSO-treated mock control (vector only) cells.

Thus far, we demonstrated that PKA phosphorylates EMSY's T207 phospho-site and that PKA expression levels have an effect on EMSY phosphorylation. We assessed EMSY's phosphorylation upon modulation of PKA activity. To manipulate PKA's activity, we used forskolin (a PKA activator) and H-89 (a PKA inhibitor). Forskolin activates adenyl cyclase, an enzyme that increases intracellular levels of c-AMP, resulting in activation of c-AMP-dependent kinases such as PKA. The PKA inhibitor H-89 inhibits the PKA catalytic subunit by competing for the ATP-binding site within PKA's catalytic pocket. EMSY-overexpressing 293T cells were treated with either forskolin or H-89 for 24 h. Following immunoprecipatation with V5 antibody, EMSY was probed using the PKA-phosphosubstrate antibody to measure PKA-targeted EMSY phosphorylation (Figure 5A). In the cells treated with forskolin, levels of EMSY phosphorylation increased, suggesting that activation of PKA increases EMSY phosphorylation. In contrast, upon PKA inhibition, EMSY phosphorylation levels were reduced. These data suggest that manipulation of PKA activity can indeed affect PKA-targeted EMSY phosphorylation.

We demonstrated that the EMSY T207 phospho-site is required for EMSY overexpression-driven HDR impairment and that PKA targets this phospho-site. Next, we explored the effects of PKA activation and inhibition on HDR impairment in EMSY-overexpressing cells. We treated EMSY-overexpressing OVCAR8^DR-GFP^ cells with either forskolin or H-89 (Figure 5B). The cells were co-electroporated with I-SceI and either control vector (mock) or EMSY-expressing plasmid, and simultaneously treated with either forskolin or H-89. The cells were incubated for 24 h to allow repair of damaged DNA. GFP-positive cells were then quantified with flow cytometry. Longer incubation was not possible due to toxicity of the drugs in electroporated cells. The differences in HDR activity (shown in Figures 1 and 2) are seen 48 h after DNA damage induction, which may explain the more prominent effect compared with the data presented in Figure 5B. In DMSO-treated cells, EMSY overexpression reduced HDR activity by ~13%, while forskolin treatment of EMSY-overexpressing cells reduced HDR activity by ~21% compared with control cells (mock, DMSO-treated). Forskolin alone did not have an impact on HDR activity. Compared with EMSY-overexpressing DMSO-treated cells, EMSY-overexpressing forskolin-treated cells demonstrated further reduction in HDR activity by ~10%. These data suggest that increased PKA activity may enhance EMSY overexpression-driven suppression of HDR. The cells treated with the PKA inhibitor H-89 alone showed a slight increase in HDR activity compared with the control cells. EMSY-overexpressing cells treated with H-89, however, showed HDR activity similar to the mock H-89-treated cells, suggesting that H-89 counteracts EMSY's suppressive effect on HDR activity.

### EMSY-overexpressing cells exhibit a subtle decrease in RAD51 expression

**Figure 6:**
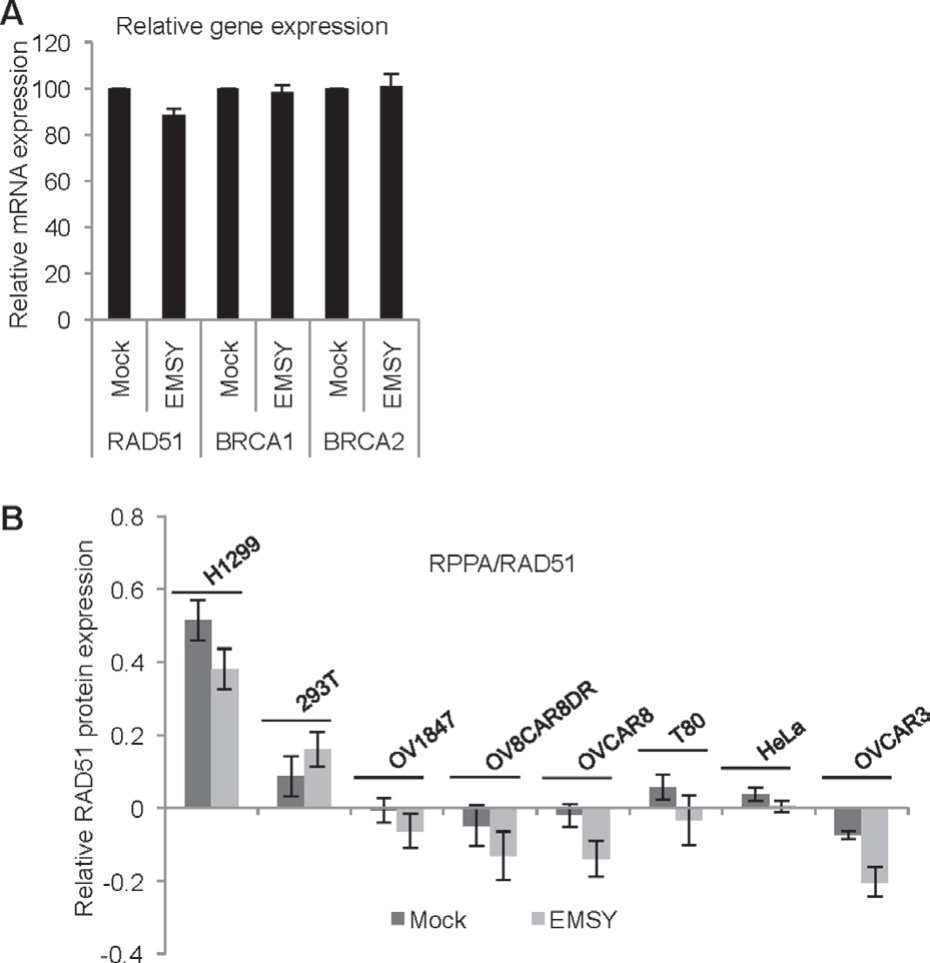
EMSY-overexpressing cells exhibit a subtle decrease in RAD51 expression (**A**) RT-PCR in EMSY over-expressing OVCAR8 cells. TaqMan probes for detecting RAD51, BRCA1 and BRCA2 were used. Values from the EMSY-overexpressing data were normalized to mock (**B**) Reverse Phase Protein Array (RPPA) in eight EMSY-overexpressing cell lines. The proteins were extracted 24 hours post-transfection and subjected to the array. Quantification of the relative RAD51 protein expression is shown.

EMSY is a well-described transcription factor. To test if expression of major players in the HDR pathway, RAD51, BRCA1 and BRCA2 decreases upon EMSY overexpression, we measured gene expression using TaqMan RT-PCR (Figure 6A). We detected a modest decrease in *RAD51* gene expression without any changes in the protein expression as measured through immunoblotting (data not shown). Given that the EMSY overexpression is short-lived and low-level, immunoblotting may not be sensitive enough to detect subtle decreases in RAD51 protein expression. To better assess protein expression changes in EMSY overexpressing cells, we employed Reverse Phase Protein Array (RPPA), a quantitative high-throughput functional proteomics assay, in eight EMSY-overexpressing cell lines (Figure 6B). Among 218 proteins and post-translational modifications (Supplementary Table 1), more than 20 are involved in DNA damage repair (e.g. AKT, ATM, ATR, BRCA2, RAD50, RAD51, PARP1, CHK1, CHK2). Among those, only RAD51 showed decreased expression in 7 out of 8 EMSY-overexpressing cell lines.

## DISCUSSION

The current work describes suppression of HDR activity in EMSY-overexpressing cells in a T207 phospho-site dependent manner. We also identified PKA as the kinase that phosphorylates this novel EMSY phospho-site. Our findings suggest that *EMSY* amplification should be considered as a BRCAness feature.

Since the discovery of EMSY, it has been suggested that *EMSY* amplification mimics the BRCA2-mutated state, resulting in defective HDR; however, the exact mechanism by which this occurs remains unknown. The proposal that EMSY plays a role in HDR stems from the observation that EMSY co-localizes with γ-H2AX foci, a marker for double-strand breaks, following ionizing irradiation in mouse embryonic fibroblast [5]. However, the authors were unable to recapitulate this in human cells, and others have not confirmed this observation. It has also been demonstrated that overexpression of a truncated form of EMSY in human mammary epithelial cells produces a chromosome instability phenotype similar to what has been seen in BRCA2-deficient cells [14]. These and subsequent studies propose *EMSY* amplification as a mechanism of BRCA2 pathway inactivation in sporadic breast and ovarian cancers [23–25]. More recent work challenges this hypothesis, suggesting that cell lines with *EMSY* amplification do not have higher sensitivity to DNA-damaging agents compared with cell lines without amplification [4]. However, these studies utilized various cell lines, which introduces issues of genetic heterogeneity that can be circumvented through the use of an isogenic background.

A major limitation in studying EMSY has been the inability to overexpress the full-length protein. Therefore, a number of non-exhaustive studies have attempted to address EMSY's role in HDR using a truncated N-terminal EMSY [5, 14, 15]. We successfully surmounted this limitation through transient full-length EMSY overexpression that peaked at ~12 h and declined by ~72 h post-transfection. Because of this short activation period, we assessed HDR activity using the DR-GFP assay 48 h post-transfection. Levels in these overexpressed EMSY cells were similar to levels of the endogenous protein, with approximately double the expression of mock controls. Therefore, the relatively modest decrease of HDR activity in EMSY-overexpressing cells, especially in U2OS^DR-GFP^ and H1299^DR-GFP^, is likely a reflection of the short-lived and low-level EMSY overexpression. We did not observe any significant changes in HDR activity in the *EMSY*-amplified OVCAR3 cells depleted of EMSY. This may suggest that HDR activity changes only upon forced EMSY over-expression. Also, EMSY knockdown does not result in a complete EMSY loss, so it may be that the residual EMSY is sufficient to maintain HDR activity unchanged.

Recently, it has been described that AKT1 phosphorylates EMSY at the S209-phospho-site, suppressing EMSY's ability to bind promoters of targeted genes [18]. Several studies have suggested a direct relationship between AKT1 and HDR [21, 22, 26, 27]. This prompted us to explore the possibility that AKT-targeted EMSY phosphorylation may affect EMSY overexpression-driven suppression of HDR. The DR-GFP assay suggested that the S209-phospho-site has less of an impact than the T207-phospho-site in HDR suppression of EMSY-overexpressing cells.

Mutant T207A and double-mutant T207AS209A overexpression demonstrated reversal of EMSY's effect on HDR suppression regardless of the source of DNA damage (Sce-I cleavage, irradiation, or CTP treatment). These data suggest that the T207 phospho-site is essential for EMSY overexpression-driven suppression of HDR activity. We identified PKA as a kinase that targets the T207 phospho-site. PKA is a cAMP-dependent serine/threonine protein kinase involved in the control of multiple cellular processes [28]. A growing body of evidence suggests contribution of PKA signaling in cancer progression [29–31]. PKA has recently been associated with the aggressive phenotype of ovarian cancer cells [32]. TCGA studies indicate that *PRKACA*, a gene that encodes PKA's catalytic subunit, is amplified in 15% of ovarian tumors, in addition to the known amplification of *EMSY* in 11% of cases (Supplementary Figure 5).

Our data demonstrate that manipulation of PKA expression and its activity in cells modulates EMSY phosphorylation. Although this suggests that EMSY is targeted by PKA, we cannot conclude that there are no other kinases that also target EMSY, possibly at the same T207 phospho-site, since the consensus sequence is conserved and recognized by multiple kinases. We tested whether PKA and EMSY co-overexpression further enhances EMSY overexpression-driven suppression of HDR in OVCAR8^DR-GFP^ cells. This attempt failed, since co-expression of the two proteins was toxic to the cells. As an alternative approach, we treated EMSY-overexpressing OVCAR8^DR-GFP^ cells with either forskolin (PKA activator) or H-89 (PKA inhibitor) and measured their impact on HDR activity. Although the data from forskolin treatment suggest a trend towards enhanced EMSY overexpression-driven impairment of HDR, cAMP also interacts with other signaling pathways that may influence HDR. EMSY-overexpressing cells treated with H-89 showed HDR activity similar to the mock-treated cells, suggesting that H-89 may counteract EMSY's suppressive effect on HDR.

The long-standing hypothesis has been that EMSY suppresses HDR in a BRCA2-dependent manner. This stems from the work that initially described EMSY as a BRCA2-interacting protein [5]. Although non-exhaustive, several attempts have been made to prove this hypothesis. Recent work suggests that truncated EMSY overexpression disrupts the RAD51/BRCA2 complex [15]; however, data from these studies do not elucidate how this is achieved. To test if full-length EMSY overexpression-driven HDR suppression is BRCA2-interaction dependent, we employed the DR-GFP assay in OVCAR8^DR-GFP^ cells. We compared HDR activity in cells overexpressing the full-length, truncated N-terminal and C-terminal EMSY (Supplementary Figure 6). Overexpression of C-terminal EMSY led to a greater decrease in HDR activity than N-terminal EMSY. Given that C-terminal EMSY lacks the ENT domain that is thought to interact with BRCA2, this result would be unexpected if EMSY's impact on HDR is BRCA2 interaction-dependent. To explore the possibility that C-terminal EMSY might also interact with BRCA2, immunoprecipitation experiments were performed and showed no interaction. Closer re-examination of EMSY-BRCA2 interaction at endogenous levels also failed to confirm that these two proteins interact. This suggests that BRCA2 and EMSY do not interact directly or that the interaction is too weak to be detected in our hands. It is likely that EMSY overexpression decreases HDR activity in a BRCA2-independent manner. BRCA2 has a major role in displacement of replication protein A (RPA) from DNA and upload of RAD51 [33]. We observed no changes in RPA32 upon EMSY overexpression (Supplementary Figure 7). The lack of increase in RPA32 foci upon EMSY overexpression supports the BRCA2-indepenent effect of EMSY on HDR.

One possible mechanism of BRCA2-independent EMSY-driven suppression of HDR could be through EMSY's transcriptional activity. EMSY is also described as an oncogene that regulates target genes in a BRCA2 interaction-independent manner [34]. Therefore, EMSY may impact HDR activity by modulating the expression of HDR-target genes. The *RAD51* gene expression and RPPA data presented here may suggest that EMSY could regulate transcriptional activity of *RAD51*. Stable and more robust EMSY overexpression and chromatin-related studies would be required to confirm this conjecture.

Collectively, our data suggest that EMSY overexpression may be a BRCAness feature. BRCAness is defined as a phenocopy of *BRCA1* or *BRCA2* mutation, where a gene alteration results in impaired HDR in the absence of germline *BRCA1* or *BRCA2* mutations [35]. It is clearly documented that patients bearing *BRCA*-mutant tumors respond well to HDR-targeted therapies such as poly (ADP-ribose) polymerase (PARP) inhibitors [36]. Recently, it has been proposed that BRCAness cancers may also respond favorably to PARP inhibitors [37]. However, BRCAness cancers are still less sensitive to PARP inhibition than *BRCA*-mutant cancers [38], possibly due to only a partial impairment of DNA damage repair, as seen in EMSY-overexpressing cells. We suggest that an increase in EMSY's T207 phosphorylation in patients bearing *EMSY*-amplified tumors could enhance BRCAness and render these patients more sensitive to PARP inhibition, a supposition that will require additional evidence in laboratory experiments and clinical trials.

## MATERIALS AND METHODS

### Cell lines and drug preparation

S2OS^DR-GFP^ and H1299^DR-GFP^ cells were established as previously described [19]. OVCAR8^DR-GFP^ cells were kindly gifted by Dr. Larry M Karnitz (Mayo Clinic College of Medicine). Wild type OVCAR8, HEK-293T and OVCAR3 cells were obtained from American Type Culture Collection (ATCC). H1299^DR-GFP^, OVCAR8^DR-GFP^, OVCAR3 and wild type OVCAR8 cells were cultured in RPMI media supplemented with 10% fetal calf serum (FCS). U2OS^DR-GFP^ and HEK-293T cells were cultured in Dulbecco's Modified Eagle's Medium (DMEM) supplemented with 10% FCS. All cells were maintained under standard conditions. All cell lines were authenticated and tested negative for the presence of *Mycoplasma*. Forskolin (#sc-3562) and H-89 (#sc-3537) were obtained from Santa Cruz Biotechnology (Santa Cruz, CA, USA). For both drugs, the stock solutions were made in dimethyl sulfoxide (DMSO) at 10 mM and stored at −80°C. For the cell treatments, 10 μM forskolin and 5 μM H-89 final concentrations were used.

### Plasmid constructs and EMSY mutants

Full-length EMSY cDNA was cloned into pENTR/D-TOPO entry vector using Gateway System (#K2400-20) from Invitrogen (Carlsbad, CA, USA). Subsequently, EMSY was sub-cloned into pcDNA3.1/nV5-DEST expression vector using Invitrogen's Gateway Vector Pack (#12290-010) to create V5-tagged pcDNA3.1/nV5-DEST-EMSY expression plasmid. This plasmid was then used to create all EMSY mutants. The mutants were created by site-directed mutagenesis using primers with specific point mutations. The validation of mutants by Sanger sequencing is shown in Supplementary Figure 1. For purposes of the *in vitro* kinase assays, wild type and mutant EMSY cDNAs (coding for N-terminal EMSY aa1-aa430) were sub-cloned into pET101/D-TOPO bacterial expression vector (Invitrogen; #K102-01) with N-terminal 6xHis tag, allowing for the protein purification on nickel beads. PKA construct cloned into pCMV6-Entry expression vector (#RC220877) was obtained from OriGene Technologies (Rockville, MD, USA). The construct contains in-frame C-terminal DDK-tag, allowing for the use of anti-FLAG antibody for the detection of the ectopically expressed PKA.

### DR-GFP assay

U2OS^DR-GFP^ (1.5×10^6^), H1299^DR-GFP^ (1×10^6^), and OVCAR8^DR-GFP^ cells (2×10^6^) were co-transfected by electroporation using Nucleofector from Amaxa Biosystems (Cologne, Germany) with 2 μg pCMV-I-SceI and 10 μg of either pcDNA3.1/nV5-DEST-EMSY (wild type or mutants) or empty vector as a mock control. The cells were harvested 48 h post-transfection, and percentages of GFP-positive cells were determined by flow cytometry (FACSCalibur; Becton Dickinson, Franklin Lakes, NJ, USA).

### RAD51 and RPA32 foci assays

OVCAR8 cells were transfected by electroporation with 10 μg of pcDNA3.1/nV5-DEST-V5-EMSY, wild type or mutants. The next day, DNA damage was induced by treating the cells either with 4 μM CPT or irradiation (10 Gy) with a Mark1 generator. Four hours after CPT treatment and 8 h after irradiation, cells were fixed and permeabilized for 15 min at room temperature with a 4% paraformaldehyde phosphate-buffered saline (PBS) solution supplemented with 0.1% Triton X-100. Cells were then washed and stained overnight at 4°C with anti-RAD51 (#sc-8349, Santa Cruz Biotechnologies) antibody. Cells were then stained with secondary antibody Alexa Fluor 488-conugated chicken anti-rabbit IgG (#A-21441, Invitrogen) at 1:1000 dilution for 1 h at room temperature. After washings, the slides were prepared using vecta-shield mounting medium containing 4′,6-diamidino-2-phenylindole (DAPI) (Vector Laboratories, Burlingame, CA), and images were obtained using a Leica-Upright SP-5 TCS confocal microscope. Cells with five or more foci were counted as RAD51-foci-positive. OVCAR3 cells depleted for EMSY were treated with CPT and assessed for RAD51 foci as described above. The RPA32 foci assessment with anti-RPA32 (#ab217, Abcam) antibody was done using the same protocol as described above for the RAD51 foci assessment.

### Immunoblotting and V5-immunoprecipitations

To prepare whole cell lysates, cells were washed twice in ice-cold PBS, and lysed with radioimmunoprecipitation assay (RIPA) buffer (sc-24948, Santa Cruz Biotechnology) supplemented with protease/phosphatase inhibitor cocktail (#88668; Pierce). Protein concentration was measured by Bradford assay (#500-0006, Bio-Rad Laboratories, Hercules, CA, USA) and boiled at 95°C for 5 min in Laemmli sample buffer (#S3401, Sigma-Aldrich, St. Louis, MO, USA). Proteins were then resolved by SDS-PAGE electrophoresis, transferred on nitrocellulose, and blotted with corresponding antibodies. Anti-V5 antibody was from Invitrogen (#R960-25, dilution 1:1000). Anti-FLAG antibody was from Sigma-Aldrich (#F1804; dilution 1:1000). Anti-AKT-phosphosubstrate (#9611; dilution 1:1000) and PKA-phosphosubstrate (#9621; dilution 1:100) antibodies were from Cell Signaling (Beverly, MA, USA). Anti-β-Actin (#sc-69879: dilution 1:10000) was from Santa Cruz Biotechnology.

For V5-immunoprecipitation purposes, 293T cells were transfected by electroporation (10μg EMSY plasmids and/or 2 μg PKA plasmid), and 24 h later the protein lysates were prepared using NETN buffer (20 mM Tris pH8, 100 mM NaCl, 0.5 mM EDTA and 1% NP-40). 20 μL (v/v) of anti-V5 agarose affinity gel (Sigma-Aldrich, #A7345) was added to the lysates and incubated for 2 h in the cold room. The agarose was washed three times in 1mL NETN buffer, 50 μL Laemmli sample buffer was added to pelleted agarose, and the samples were boiled at 95°C for 5 min. Immunoblots were performed as above. Simultaneous PKA knockdown and EMSY overexpression were done by co-electroporating siRNAs (2 μL of 10 μM stock) and plasmids (10μg). Control siRNA (#D-001810-10-05) and PKA-targeting siRNA (#L-004649-00-0005) were from Dharmacon (Lafayette, CO, USA).

### RNA extraction and quantitative real-time PCR

Total RNA was isolated from mock and EMSY-overexpressing OVCAR8 cell pellets using the mirVana miRNA isolation kit (Ambion). For analysis of mRNA expression, cDNA was synthesized from 2 μg of RNA with the Applied Biosystems™ High-Capacity cDNA Reverse Transcription Kit (Applied Biosystems). PCR was performed on a ViiA^™^ 7 Real-Time PCR System (Applied Biosystems) using TaqMan^®^ Gene Expression Assays for RAD51, BRCA1 and BRCA2. Expression levels were normalized to GAPDH.

### Reverse phase protein array (RPPA)

RPPA was done in collaboration with the RPPA Core Facility at MD Anderson Cancer Center (https://www.mdanderson.org/research/research-resources/core-facilities/functional-proteomics-rppa-core.html). The protein lysates were prepared according to the Core's instructions and send to them for the profiling. Briefly, eight cell lines (H1299, 293T, HeLa, OVCAR3, OVCAR8, OV1847, OVCAR8^DR-GFP^ and T80) were transfected with either empty vector (mock) or EMSY plasmid and 24 hr later the cells were pelleted. Proteins were extracted by adding RPPA extraction buffer (50mM Hepes pH 7.3; 150 mM NaCl; 1.5 mM MgCl_2_; 1 mM EGTA; 10 mM Na-pyrophosphate; 1 mM Na_3_VO_4_; 100 mM NaF; 1% Triton; 10% Glycerol) to the cell pellets and incubating on ice for 20 minutes. Protein concentrations were measured by the Bradford assay. 40 μg of protein lysates was submitted to the Core for the analysis. All samples were done in triplicates. The Core stained 243 slides for 218 unique antibodies (Supplementary Table 1) which were analyzed on Array-Pro then by supercurve R ×64 2.15.1. There were 14 sets of replicated antibodies and 3 negative controls for secondary antibodies among 243 slides. QC test were performed for each antibody staining (slide). All the data points were normalized for protein loading, transformed to linear values and subsequently transformed to Log2 values.

### Bacteria-expressed protein purification and *in vitro* kinase assay

To express and purify N-terminal EMSY wild type and mutants in bacteria, we utilized Invitrogen's Champion pET Directional TOPO Expression Kit (#K102-01). N-terminal 6xHis-V5 tagged EMSY constructs were transformed in BL21 Star *E. coli*. 250 μL SOC media was added and after 30 minute incubation at 37°C, the transformed bacteria was added to 10 mL terrific broth (TB) supplemented with 100ug/mL ampicillin and incubated over night at 37°C. 250 μL of overnight culture was added to 5 mL TB and incubated at 37°C until cells reached ~0.5 OD^600^. 1 mM Isopropyl β-D-1-thiogalactopyranoside (IPTG) was added to induce protein expression. After 2 h of incubation at 37°C, the cells were pelleted at 3000 × g for 5 min and lysed with 1 × Native Binding Buffer supplemented with lysozyme for 30 min on ice. Subsequently, the cells were sonicated and pelleted at 3000 × g for 15 min. The lysates were then incubated with 100 μL (v/v) Ni-NTA Magnetic Agarose nickel beads (#36111) from QIAGEN (Valencia, CA, USA) for 2 h in the cold room. The beads were washed using magnetic stand with 1 mL Native Wash buffer supplemented with 20 mM imidazole. Purified EMSY proteins were then eluted in 100 μL elution buffer supplemented with 250 mM imidazole and stored at 4°C.

For the purposes of *in vitro* kinase assays, recombinant kinases AKT1 (#31145), AKT2 (#31146), AKT3 (#31147), S6K (#31193), and PKA (#31158) were purchased from Active Motif (Carlsbad, CA, USA). For the 20 μL kinase reaction, 10μg of a kinase and 5 μL of eluted proteins were mixed in the kinase buffer (Cell Signaling, #9802) and incubated for 1 h at room temperature. The reaction was stopped by adding 20 μL Laemmli sample buffer and boiling for 5 minutes at 95°C. The proteins were resolved on 4–20% SDS-PAGE and immunoblotted with AKT-phosphosubstrate or PKA-phosphosubstrate antibodies as described above.

## SUPPLEMENTARY MATERIALS FIGURES AND TABLES




