# Supplementary material for: The EMSY threonine 207 phospho-site is required for EMSY-driven suppression of DNA damage repair
Source: Oncotarget. 2017 Jan 13;8(8):13792–804. doi: 10.18632/oncotarget.14637 (PMC5355139; doi:10.18632/oncotarget.14637)
Supplement: Supplementary file 1 [file oncotarget-08-13792-s001.pdf]

The EMSY threonine 207 phospho-site is required for EMSY-driven suppression of DNA damage repair

Supplementary Materials

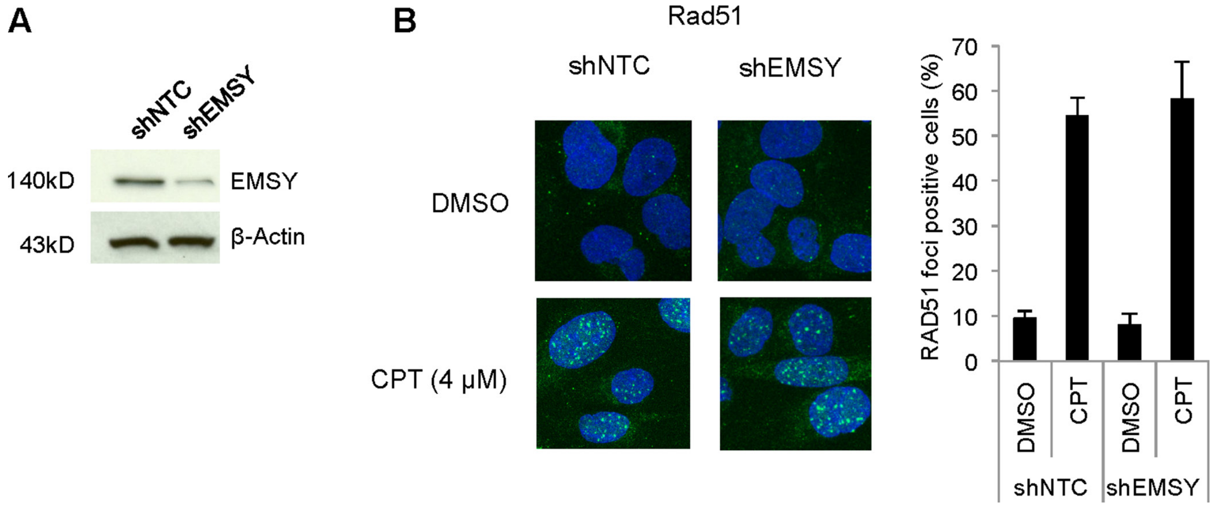

Supplementary Figure 1: RAD51 foci formation assessment in EMSY KD OVCAR3 cells. (**A**) Western blot showing OVCAR3 cells stably depleted for EMSY with shRNA (shEMSY). shNTC (non-template control) was used as a negative control. (**B**) RAD51 foci formation in CPT treated cells with representative images and quantification.

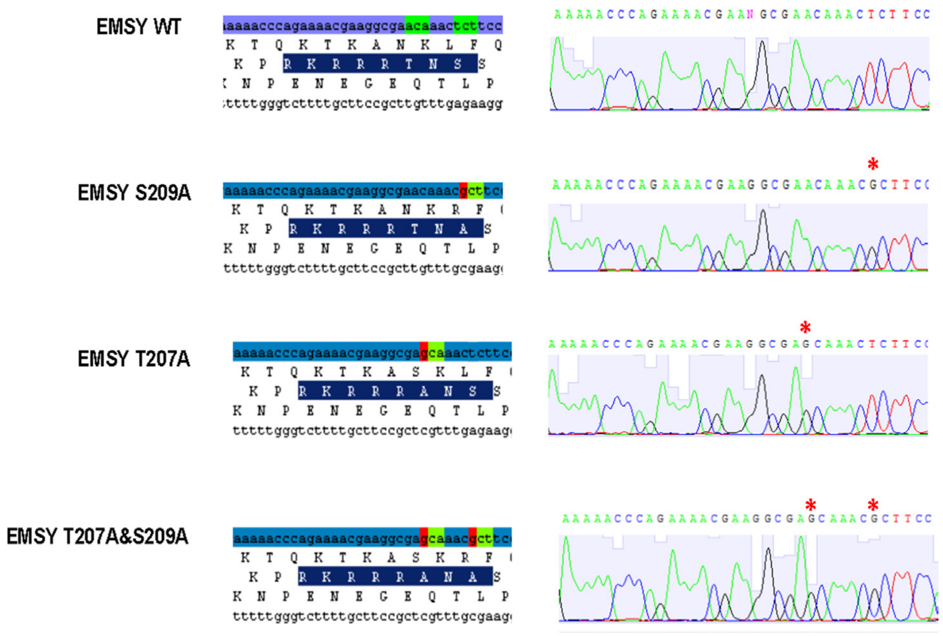

Supplementary Figure 2: Confirmation of EMSY mutations with Sanger sequencing.

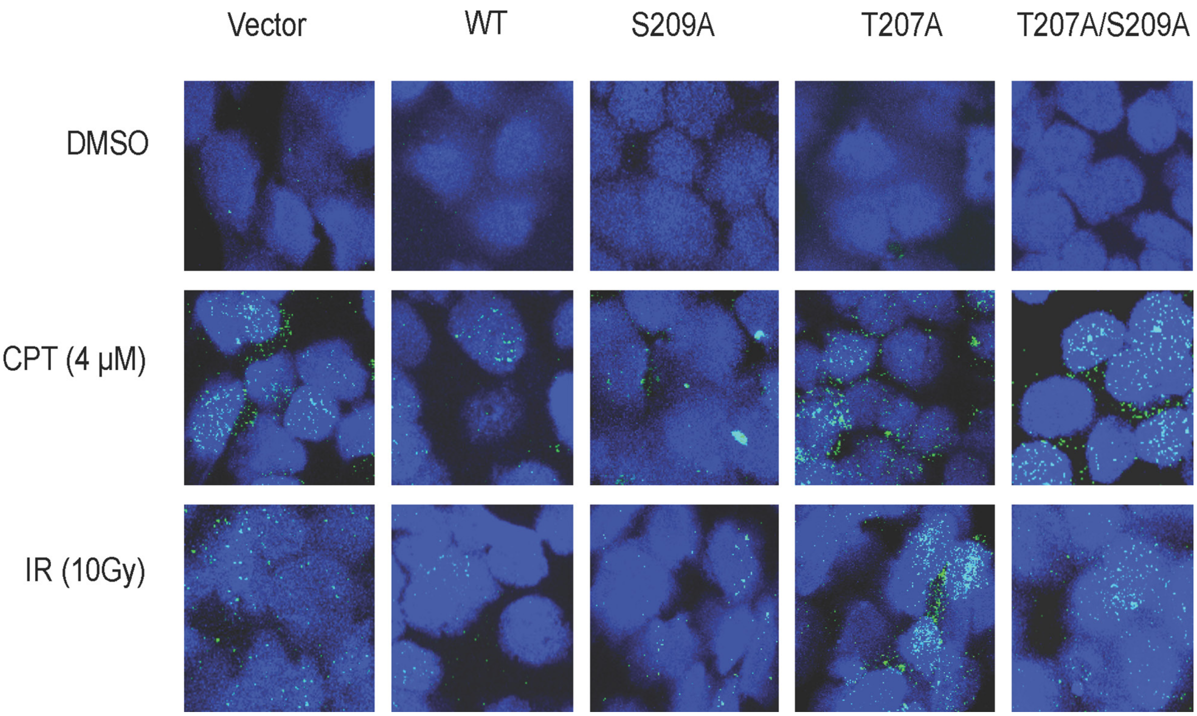

Supplementary Figure 3: RAD51 foci formation immunocytochemistry in OVCAR8 cells overexpressing wild type and mutant EMSY. Cells were treated with either 4 µM camptothecin (CPT) or irradiation (10 Gy). DMSO-treated cells were used as a control. Cells with five or more RAD51 foci were counted as positive.

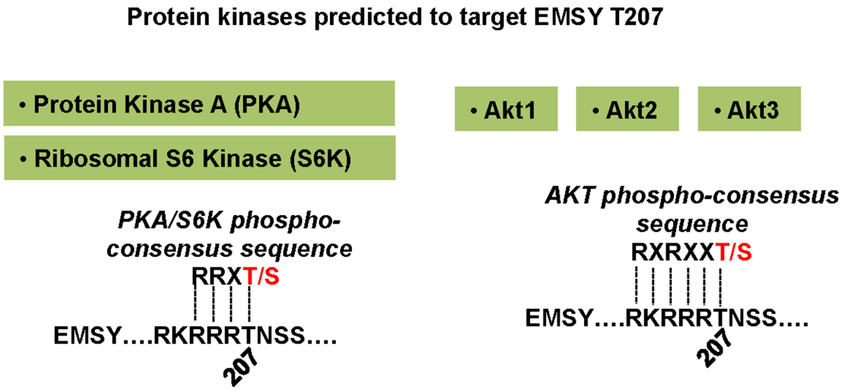

Supplementary Figure 4: Protein kinases predicted to target the EMSY T207 phospho-site by group-based prediction system for protein kinase-specific prediction (http://gps.biocuckoo.org). Consensus sequences are shown below the corresponding kinases.

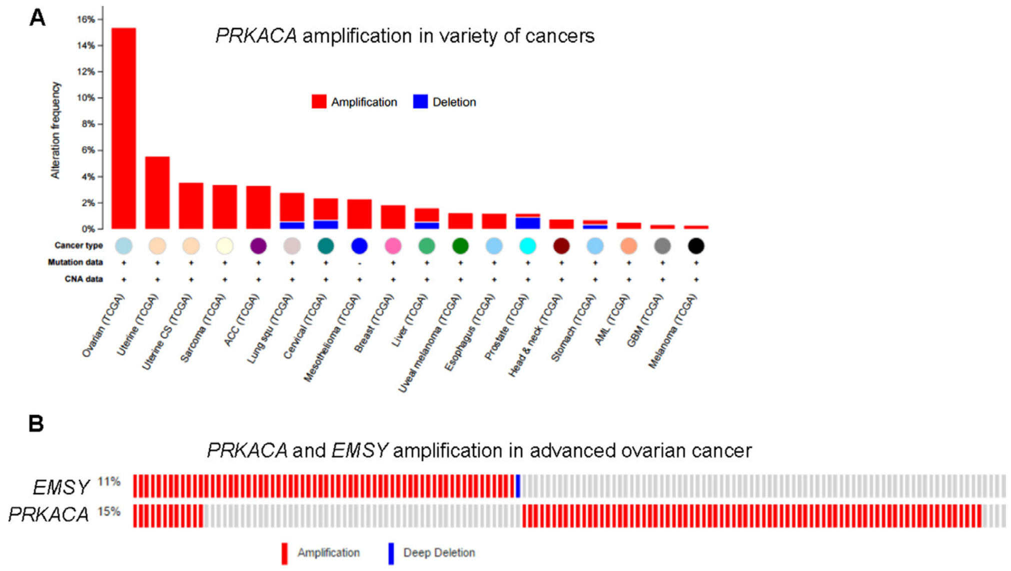

Supplementary Figure 5: PRKACA, encoding PKA, amplification in advanced ovarian carcinoma (source: cbioportal.mskcc.org). (**A**) PRKACA amplification is most frequent in advanced ovarian carcinoma. (**B**) Frequent amplification of both PRKACA and EMSY in advanced ovarian carcinoma.

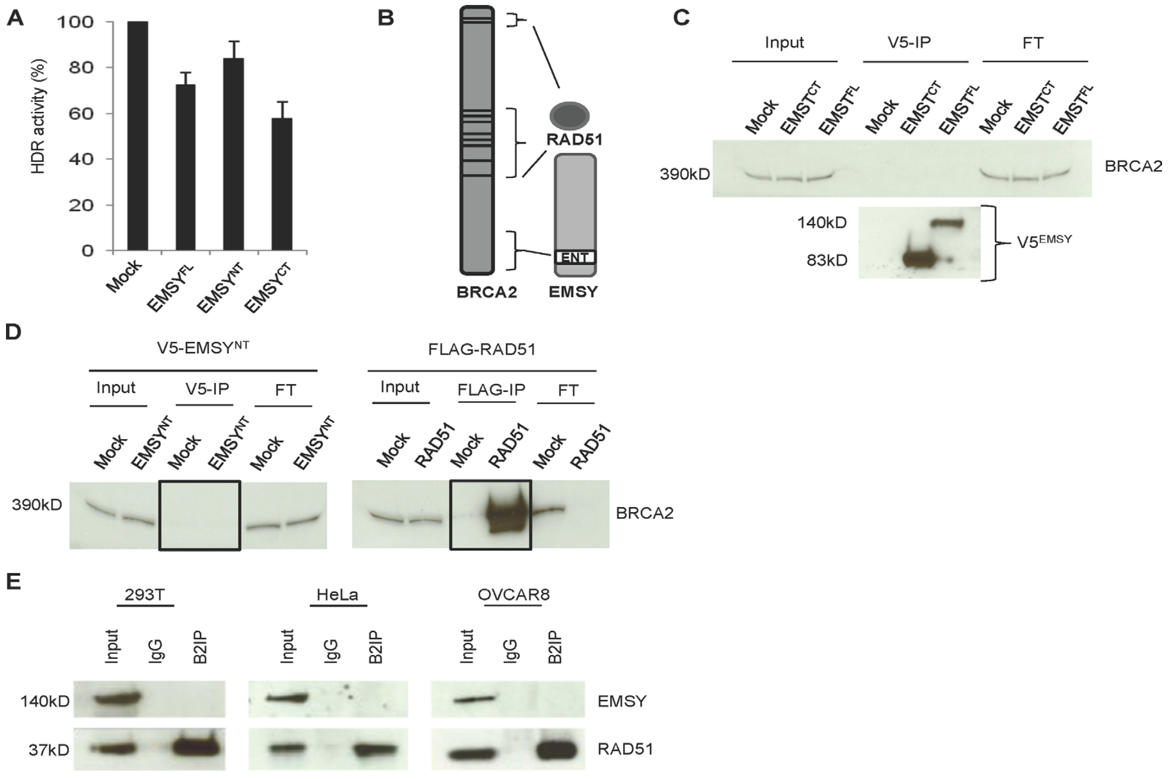

Supplementary Figure 6: EMSY does not interact with BRCA2. (**A**) The DR-GFP assay in cells overexpressing the full-length, N-terminal and C-terminal EMSY. HDR activity is normalized to mock controls (empty vector-transfected cells). (**B**) Schematic representation of BRCA2 interactions with RAD51 and EMSY. (**C**) Immunoprecipitation with the V5 antibody in cells overexpressing the full-length or C-terminal EMSY. The blots are probed with BRCA2 antibody. The flow-through (FT) represents the lysates after the immunoprecipitation (IP) reaction. Overexpressed EMSY is detected with the V5 antibody (V5EMSY). (**D**) IP with the V5 and FLAG antibodies in cells overexpressing V5-tagged N-terminal EMSY or FLAG-tagged RAD51 (positive control). FLAG-IP indicates successful IP; BRCA2 is co-immunoprecipitated with the overexpressed RAD51. (**E**) Endogenous IP in 293T, HeLa, and OVCAR8 cell lines with BRCA2 antibody (B2IP). Non-specific IgG served as an IP control. RAD51 served as a positive control.

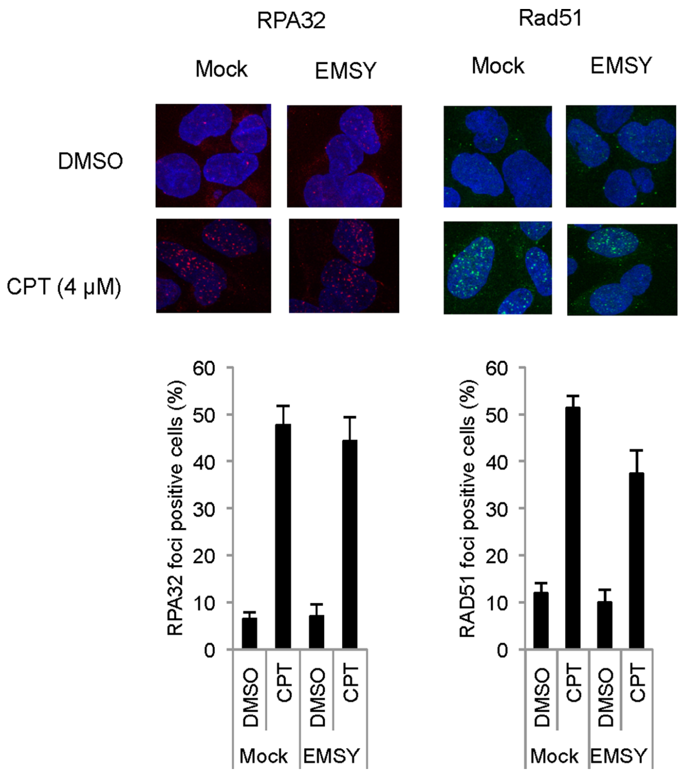

Supplementary Figure 7: No change in RPA32 foci formation upon EMSY overexpression. RPA32 and RAD51 foci formation in CPT-treated OVCAR8 cells. Quantification of RAD51-positive cells is shown below the images.

Supplementary Table 1: RPPA antibody list. See_Supplementary_Table_1
